# Discovery, mode of action and secretion of *Burkholderia* sensu lato key antimicrobial specialised metabolites

**DOI:** 10.1016/j.tcsw.2022.100081

**Published:** 2022-10-13

**Authors:** Yoana D. Petrova, Eshwar Mahenthiralingam

**Affiliations:** School of Biosciences, Cardiff University, Cardiff, UK

**Keywords:** *Burkholderia*, Specialised metabolite, Antimicrobial, Mechanism of action, Secretion

## Abstract

*Burkholderia* sensu lato bacteria have genomes rich in biosynthetic gene clusters (BGCs) encoding for multiple bioactive specialised metabolites. Diverse classes of antimicrobial natural products have been isolated from *Burkholderia*, including polyynes, shikimate pathway derivatives, polyketides, non-ribosomal peptides and hybrid polyketide non-ribosomal peptides. We highlight examples of *Burkholderia* metabolites, overviewing their biosynthesis, bioactivity, mechanisms of action and secretion.

## 1 Introduction

The *Burkholderia* genus encompass Gram-negative bacteria that are part of the *Burkholderia* sensu lato multi-genus complex, which includes *Paraburkholderia*, *Trinickia*, *Cabballeronia*, *Mycetohabitans*, *Robbsia* and *Pararobissa* (Mullins & Mahenthiralingam, 2021). The *Burkholderia* clade encompasses the *Burkholderia cepacia* complex (Bcc), *Burkholderia pseudomallei* group, and a plant pathogen group characterised by *Burkholderia gladioli* (Mullins & Mahenthiralingam, 2021). *Burkholderia* species display extensive phenotypic plasticity, allowing them to occupy multiple niches ranging from the plant root to the cystic fibrosis lung (Kunakom & Eustaquio, 2019). The ability of *Burkholderia* to adapt to different environments is attributed to their large genomes with complex organisation into multiple replicons (Kunakom & Eustaquio, 2019). *In silico* analysis of > 4000 *Burkholderia* sensu lato genomes revealed large average genome size (7.57 Mbp) with high GC content (66.73 %), with 14.6 % of the *Burkholderia* genomes comprising biosynthetic gene clusters (BGCs) dedicated to specialised metabolite biosynthesis (Mullins & Mahenthiralingam, 2021). The extensive potential of *Burkholderia* species to produce specialised metabolites is being increasingly exploited for the discovery of natural products with antimicrobial properties for the use in clinic, agriculture and biotechnology (Kunakom & Eustaquio, 2019).

## 2 Examples of bioactive *Burkholderia* specialised metabolites

**Fig. 1:**
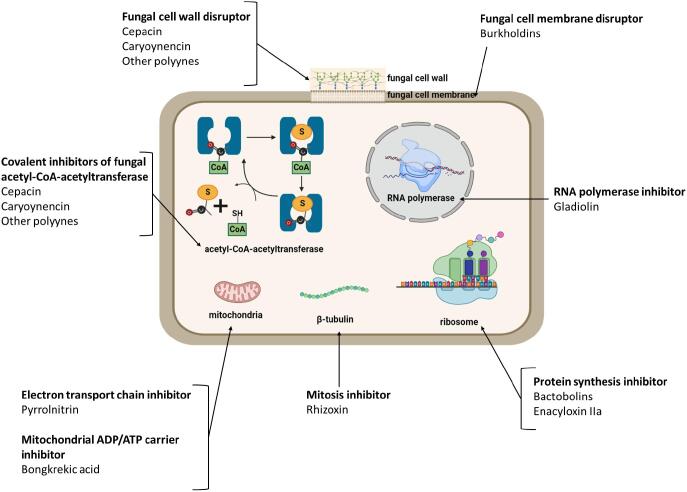
**Mechanism of action and cellular targets of selected bioactive *Burkholderia* specialised metabolites.** The diagram shows the cellular targets and mechanism of action specialised metabolites with antimicrobial properties produced by *Burkholderia* bacteria. The illustration was created with BioRender.com.

Selected antimicrobial specialised metabolites produced by the *Burkholderia* genus are outlined with their structure, class and bioactivity spectrum (Table 1) and cellular target(s) illustrated (Fig. 1).

**Table 1:** Structure and properties of selected specialised metabolites produced by *Burkholderia* sensu lato species.

**Specialised metabolite and structure**	**Metabolite class**	**Secretion mechanism**	**Bioactivity**	**Producers**	**Reference**
**Pyrrolnitrin**	Shikimate pathway derivative	Putative multidrug transporter	Fungi; oomycetes; Gram-positive bacteria;	*B. pyrrocina* *B. vietnamiensis* *B. ubonensis B. lata B. cepacia B. cenocepacia B. ambifaria B. pseudomallei B. thailandensis*	(El-Banna and Winkelmann, 1998, Kirner et al., 1998, Costa et al., 2009)
**Cepacin A**	Polyyne	MFS transporter	*Staphylococci*; Gram-positive bacteria; *Globisporangium ultimum*	*B. cepacia* *B. ambifaria* *B. diffusa* *B. vietnamiensis*	(Parker et al., 1984, Mullins et al., 2019, Lin et al., 2022)
**Enacyloxin IIa**	Hybrid polyketide non-ribosomal peptide	MATE transporter upregulated during biosynthesis	Gram-negative bacteria	*B. ambifaria* *B. gladioli*	(Mahenthiralingam et al., 2011, Jones et al., 2021)
**Gladiolin**	Polyketide	Multidrug transporter (MATE family)	*Mycobacteria tuberculosis;* *Candida albicans;* Gram-positive	*B. gladioli*	(Song *et al.*, 2017)
**Caryoynencin**	Polyyne	RND efflux system; HlyD secretion protein	Fungi; Gram-positive bacteria; Oomycetes	*B. gladioli T. caryophylli*	(Kusumi et al., 1987, Ross et al., 2014, Lin et al., 2022)
**Burkholdins**	Non-ribosomal peptide	Putative cyclic peptide transporter	Fungi	*B. ambifaria B. cepacia B. contaminans*	(Tawfik *et al.*, 2010, Lin et al., 2012, Thomson & Dennis, 2012)
**Bactobolin**	Hybrid polyketide non-ribosomal peptide	Bcr/CflA family efflux transporter	Gram-positive and Gram-negative bacteria	*B. thailandensis B. ambifaria B. pseudomallei B. mallei*	(Seyedsayamdost et al., 2010, Chandler et al., 2012)
**Bongkrekic acid**	Polyketide	Unknown	Eukaryotic cells	*B. gladioli*	(Moebius et al., 2012)
**Rhizoxin**	Hybrid polyketide non-ribosomal peptide	MFS transporter; ABC transporter	Eukaryotic cells	*M. rhizoxina* endosymbiont of *Rhizopus microsporus*	(Partida-Martinez and Hertweck, 2005, Partida-Martinez and Hertweck, 2007)

ABC = ATP-binding cassette superfamily, MFS = major facilitator superfamily, RND = resistance-nodulation-cell-division superfamily; MATE = multidrug and toxin extrusion transporter.

### 2.1 Polyynes

Bacterial polyynes are characterised by alternating single and triple carbon–carbon bonds, with a terminal alkyne, which makes them chemically unstable and challenging to purify (Lin et al., 2022, Petrova et al., 2022). The core biosynthetic genes in the bacterial polyyne gene clusters, associated with alkyne biosynthesis, are a fatty acyl-AMP ligase, a fatty acid desaturase and an acyl carrier protein (Mullins et al., 2021). The two additional fatty acid desaturases present in the polyyne BGC core, the thioesterase and the rubredoxin genes, are also identified as being specifically conserved for biosynthesis (Mullins et al., 2021).

*Burkholderia* produce two polyynes. Cepacin is encoded by a 13 kbp BGC found in multiple Bcc species (Table 1) (Parker et al., 1984, Mullins et al., 2019), and caryoynencin (11 kbp BGC) produced by *B. gladioli* and also *Trinickia* (formerly *Burkholderia*) *caryophilli* (Table 1) (Kusumi et al., 1987, Ross et al., 2014). They have a broad antimicrobial spectrum against Gram-positive bacteria, filamentous fungi and oomycetes (Table 1), which renders them of interest for biopesticide engineering (Petrova et al., 2022). Cepacin has been demonstrated to be a key metabolite in mediating the protection offered by the biopesticide *B. ambifaria* BCC0191 against oomycete damping-off disease in germinating pea seedlings (Mullins et al., 2019).

The recently elucidated mechanism of action of bacterial polyynes involves specific covalent inhibition of fungal acetyl-CoA acetyltransferase and fungal cell membrane disruption (Lin et al., 2022) (Fig. 1). *Burkholderia* polyynes also have potent activity against Gram-positive bacteria (Petrova et al., 2022) while the exact targets are not proven, it is reasonable to assume a similar mechanism of action. The export mechanism for *Burkholderia* polyynes has not yet been elucidated experimentally, but their BGCs encode efflux transporters such as an MFS transporter for cepacin BGC (Mullins et al., 2019) and an RND efflux system for caryoynencin BGC (Ross et al., 2014), which may play a role in their secretion.

### 2.2 Shikimate pathway derivatives

Pyrrolnitrin is an l-tryptophan derived bacterial metabolite produced by a small ∼ 5.8 kbp BGC consisting of four genes, *prnABCD* (Kirner et al., 1998), which is widely distributed in *Burkholderia* (Table 1). l-tryptophan is a downstream product of the shikimate pathway (Pittard & Yang, 2008), therefore pyrrolnitrin is classed as a shikimate pathway derived metabolite (Kunakom & Eustaquio, 2019). The biosynthesis of pyrrolnitrin occurs in sequential steps, starting with the chlorination of l-tryptophan by the *prnA* encoded halogenase, followed by ring rearrangement catalysed by *prnB*, further chlorination by *prnC* halogenase gene product and oxidation of the amino group to a nitro group by the *prnD* encoded oxygenase (Kirner et al., 1998).

Although the pyrrolnitrin BGCs does not contain a dedicated transporter gene, putative mutltidrug transporters have been detected in the vicinity of the cluster (Costa et al., 2009). Pyrrolnitrin production by Bcc organisms is regulated by *N*-acylhomoserine lactone (AHL)-dependent quorum sensing and enhanced by the use of glycerol as a carbon source in the growth media (Keum et al., 2009). Pyrrolnitrin acts as uncoupling agent in oxidative phosphorylation by blocking electron transfer between dehydrogenases and cytochrome components of the electron transport chain in the mitochondria (Fig. 1) (El-Banna & Winkelmann, 1998). As a result, it has a broad bioactivity against filamentous fungi and oomycetes (Table 1) (El-Banna & Winkelmann, 1998). Pyrrolnitrin also has activity against Gram-positive bacteria (Table 1), including *Streptomyces* species, which is assumed to be exerted by inhibition on bacterial growth by complexing with the cell membrane phospholipids (El-Banna & Winkelmann, 1998).

### 2.3 Polyketides

These complex *Burkholderia* specialised metabolites are synthesised by modular polyketide synthases (PK), consisting of at least four biosynthetic domains (Esmaeel et al., 2018). The acyltransferase domain catalyses the transfer of acetyl-CoA or malonyl-CoA substrate onto the acyl carrier protein domain; this is followed by a condensation reaction between the two carboxylic acids in the polyketide chain catalysed by a ketosynthase domain and the final release of the final product from the enzyme by the thioesterase domain (Esmaeel et al., 2018). Polyketide structures can be further modified by additional biosynthetic domains including ketoreductase, dehydratase, and enoyl reductase modules resulting structurally diverse natural products with diverse properties and bioactivity (Esmaeel et al., 2018).

Gladiolin, is a polyketide macrolide antibiotic encoded for by a 130 kbp BGC found in a sub-clade of *B. gladioli* strain (Jones et al., 2021), which exhibits a moderate bioactivity against clinically relevant Gram-positive bacteria (methicillin-resistant *Staphylococcus aureus* and *Enterococcus faecium*), and potent activity against isoniazid resistant *Mycobacteria tuberculosis* strains (Table 1) (Song *et al.*, 2017). The cellular target of gladiolin is bacterial RNA polymerase leading to inhibition of transcription (Fig. 1) (Song *et al.*, 2017). Gladiolin was also found to have antifungal activity against *Candida albicans*, but low toxicity when tested against ovarian cancer cell lines *in vitro* and the *Galleria mellonella* wax moth larvae model *in vivo* (Song *et al.*, 2017). Overall, gladiolin shows promise as a treatment for antibiotic resistant tuberculosis. In contrast, bongkrekic acid, another polyketide natural product encoded by clade 1*B. gladioli* strains (Jones et al., 2021), acts as a highly potent respiratory toxin in both eukaryotic and prokaryotic cells by inhibiting the electron transport chain (Table 1; Fig. 1) (Moebius et al., 2012).

### 2.4 Non-ribosomal peptides

*Burkholderia* non-ribosomal peptide (NRP) natural products are synthesised in a ribosome-independent manner by multi-domain non-ribosomal peptides synthetases (NRPSs) mega-enzymes, that can incorporate both L and D amino acids in the peptide chain (Esmaeel et al., 2018). The NRPSs contain condensation, adenylation, thiolation and thioesterase domains for the raw peptide product synthesis and additional epimerization, oxidation, and methylation modules involved in the final product modification (Esmaeel et al., 2018).

Burkholdin compounds from *B. ambifaria* and *B. cepacia*, and the nearly identical in structure occidiofungins from *B. contaminans,* are cyclic lipopeptides with potent antifungal properties (Lin et al., 2012). These NRPs exert their mechanism of action by disrupting fungal cell membranes (Fig. 1) and the membrane disruption mechanism can occur in any eukaryotic cell leading to toxicity and effects such as haemolysis during Bcc infection (Thomson & Dennis, 2012).

### 2.5 Hybrid polyketide non-ribosomal peptides

The genomes of certain *Burkholderia* also contain hybrid BGCs with both PKS and NRPS domains (Mullins & Mahenthiralingam, 2021), giving rise to hybrid PKS-NRPS products (Esmaeel et al., 2018). Enacyloxin IIa, encoded for by an unusual PKS-NRPS gene cluster with PKS modules containing both *cis-* and *trans-*acetyltransferase domains, is produced by *B. ambifaria* (Mahenthiralingam et al., 2011) and *B. gladioli* strains (Jones et al., 2021) (Table 1). Enacyloxin IIa is bioactive against several antibiotic resistant Gram-negative bacteria, including the cystic fibrosis opportunistic *Burkholderia multivorans*, and targets the elongation factor Tu of the ribosome, preventing release of aminoacyl-tRNA and therefore leading to protein synthesis inhibition (Mahenthiralingam et al., 2011) (Fig. 1).

The bactobolins are another example of hybrid PKS-NRPS metabolites produced by *Burkholderia*, with activity against both Gram-positive and Gram-negative bacteria (Table 1) (Seyedsayamdost et al., 2010). Four bactobolin isomers that share the same core structure and similar bioactivity have been characterised, but they vary in their side chains (Seyedsayamdost et al., 2010). The production of bactobolins is also quorum sensing regulated in *Burkholderia* (Seyedsayamdost, 2014) and biosynthesis is greater when grown at 30 °C compared to 37 °C (Chandler et al., 2012). The bactobolins inhibit bacterial protein synthesis by targeting the 50S ribosome-associated L2 protein (Fig. 1) (Chandler et al., 2012). Despite the potent antibacterial activity of bactobolins, their toxicity against mouse fibroblasts *in vitro* excludes their potential for clinical use (Chandler et al., 2012).

Rhizoxin is PKS-NRPS compound produced by *Mycetohabitans rhizoxina* formerly known as “*B. rhizoxina”* and now reclassified as its own genus within *Burkholderia* sensu lato (Mullins & Mahenthiralingam, 2021). *M. rhizoxina* is an endosymbiont of the *Rhizopus microsporus* fungus (Table 1) (Partida-Martinez & Hertweck, 2005). The fungus causes blight in rice seedlings attributed to the bacterial production of rhizoxin, which binds β-tubulin in eukaryotic cells leading to inhibition of mitosis (Partida-Martinez & Hertweck, 2005) (Fig. 1). It has been demonstrated that the production of rhizoxin by the endosymbiotic *B. rhizoxina* confers a survival advantage to the *R. microsporus* fungus by providing protection against protozoan and metazoan predators (Richter et al., 2022).

## 3 Conclusion

*Burkholderia* sensu stricto bacteria produce an array of structurally diverse specialised metabolites with antifungal and antibacterial properties. They represent a relatively untapped resource for the discovery of antimicrobial natural products with diverse cellular targets, and promising potential for clinical and agricultural use.

## CRediT authorship contribution statement

**Yoana D. Petrova:** Conceptualization, Visualization, Investigation, Writing – original draft, Writing – review & editing. **Eshwar Mahenthiralingam:** Conceptualization, Funding acquisition, Investigation, Supervision, Writing – review & editing.

## Declaration of Competing Interest

The authors declare that they have no known competing financial interests or personal relationships that could have appeared to influence the work reported in this paper.

## Data Availability

No data was used for the research described in the article.
